# Defining the recommended gray zone in O6-methylguanine-DNA methyltransferase promoter methylation pyrosequencing reporting: A robust, translatable method to implement new EANO guidelines

**DOI:** 10.1093/noajnl/vdaf061

**Published:** 2025-03-22

**Authors:** Polly Taylor, Gabrielle Cruickshank, Jack Wildman, George Doyle, Ed Whittaker, Sara Walker, Claire McKeeve, Claire Faulkner, Laura Yarram-Smith, Paul White, Kathreena M Kurian

**Affiliations:** Bristol Medical School, University of Bristol, UK; Bristol Medical School, University of Bristol, UK; Neurosurgery, North Bristol NHS Trust, UK; Bristol Genetics Laboratory, North Bristol NHS Trust, UK; Leverndale Hospital, NHS Greater Glasgow and Clyde, UK; School of Health and Wellbeing, University of Glasgow, UK; Beatson West of Scotland Cancer Centre, NHS Greater Glasgow and Clyde, UK; West of Scotland Centre for Genomic Medicine, NHS Greater Glasgow and Clyde, UK; Bristol Genetics Laboratory, North Bristol NHS Trust, UK; Bristol Genetics Laboratory, North Bristol NHS Trust, UK; Mathematics and Statistics Research Group, University of the West of England, UK; Bristol Medical School, University of Bristol, UK; Neuropathology, North Bristol NHS Trust, UK

**Keywords:** cutoff, glioblastoma, gray zone, MGMT methylation, pyrosequencing

## Abstract

**Background:**

The DNA repair protein O6-methylguanine-DNA methyltransferase (MGMT) may cause resistance of tumor cells to alkylating agents and is a predictive biomarker in high-grade gliomas treated with temozolomide. Recent European Association of Neuro-Oncology (EANO) guidelines recommend internal validation of MGMT methylation cutoffs and reporting of gray zone values. This study aimed to develop a method to derive a gray zone from pyrosequencing MGMT methylation data.

**Methods:**

We developed a method to find the optimal gray zone using pyrosequencing MGMT methylation values (CpG sites 72–83) from 308 glioblastoma cases with overall survival data. Each integer below the methylated threshold defined a new possible gray zone and categorization which was used as a variable in a multivariate Cox proportional hazards regression model. The optimal gray zone was selected as the option that had a statistically different survival function from the methylated and unmethylated groups, with the largest log-likelihood ratio test statistic. We applied the method to a validation cohort of 115 glioblastoma cases.

**Results:**

Our method successfully identified a gray zone in our development cohort. The following categorization gave 3 distinct survival functions: methylated ≥12% (*n* = 152 cases), gray zone 5%–12% (*n* = 43), and unmethylated <5% (*n* = 113). This categorization was better at predicting survival than the existing categorization (methylated ≥12%, unmethylated <12%). Validating our method showed a sufficient sample size and time to follow up is recommended to apply our method.

**Conclusions:**

We have developed a translatable method to identify the optimal MGMT gray zone from pyrosequencing data in line with recent EANO guidelines, to enhance clinical decision-making.

**Key Points**

- Translatable method to find the MGMT gray zone, based on overall survival and multivariate Cox regression analysis.
- Significant result when applied to pyrosequencing MGMT methylation values (CpG sites 72–83) in 308 GBM patients.

**Importance of the Study**

Recent EANO guidelines recommend reporting O6-methyl guanine methyltransferase (MGMT) methylation gray zone results and cutoff values for clinical decision-making. Different techniques to extract the methylation data can result in different MGMT methylation values, and values can also vary between laboratories using the same technique, due to factors such as equipment and biological variability. Previous studies have described methods to determine the gray zone from qMSP assay data; however, these methods cannot be readily applied to pyrosequencing data which is generated by many laboratories. Our novel method provides a simple, translatable process by which the MGMT gray zone can be derived from pyrosequencing data, thus identifying a safety margin for weakly methylated GBM patients who may potentially benefit from temozolomide.

Glioblastoma, IDH-wild type (GBM) is the most common primary brain tumor in adults, with an age-adjusted incidence ranging from approximately 0.59 to 3.69 per 100 000 population.^1^ Current first-line therapy comprises the Stupp protocol and consists of surgical resection, followed by radiotherapy with concomitant and adjuvant temozolomide chemotherapy.^2^ The median overall survival (OS) following the Stupp protocol is 15 months, and 9.9 months for patients treated with surgery and radiotherapy.^3^

Temozolomide promotes cell death by alkylating several sites within DNA, including O6 sites of guanine.^4^ Chemoresistance can be a significant problem for some GBM patients, largely due to the O6-methyl guanine methyltransferase (MGMT) repair enzyme that reverses the alkylation and methylation of guanine at the O6 position.^4^ Expression of the MGMT enzyme is related to the epigenetic silencing of the MGMT promoter, where methylation of the MGMT promoter improves response to alkylating agents like temozolomide.^5^ MGMT methylation status is predictive for IDH-wild-type GBM patients since the frequent loss of chromosome 10 in IDH-wild-type GBM means that only 1 MGMT allele at 10q26 needs to be silenced.^6^ For other glial, glioneuronal and neuronal tumor types, the predictive value of the MGMT methylation status is still to be determined.^6^

MGMT promoter methylation is present in approximately 40-50% of IDH-wild-type glioblastomas.^7^ Several techniques can be used to establish the methylation status, including methylation-specific polymerase chain reaction (MSP), quantitative (or real-time) methylation-specific PCR (qMSP), methylation-specific sequencing including pyrosequencing (PSQ), bead array, methylation-specific multiplex ligation-dependent probe amplification (MS-MLPA), PCR with methylation-sensitive high-resolution melting (PCR-HRM), co-amplification at lower denaturation temperature PCR (COLD-PCR) and digestion-based assays.^8^ However, internationally accepted consensus about the most appropriate diagnostic method for MGMT promoter status is lacking.^9^

Stupp et al classified a tumor as methylated or unmethylated using a threshold such that the probability of being methylated or unmethylated was 50%.^10^ Dunn et al defined the threshold as the average MGMT methylation in non-neoplastic brain samples (mean ± 2 SD) from pyrosequencing and MSP, which gave a cutoff of 9%.^11^ For measurements attained by qMSP, both Vlassenbroeck et al and Hegi et al used a bimodal Gaussian distribution to model the methylation data and set the threshold to be the minimum between the 2 curves.^12,13^ Hegi et al also used OS to supervise their model on MSP data, which used time-dependent receiver operating characteristic (ROC) analysis and nearest neighbor estimation to define the threshold.^13^

A review of over 20 studies on MGMT methylation pyrosequencing in glioblastoma reported that most studies derived a threshold between 8% and 10%.^14^ Nguyen et al analyzed the mean percentage methylation of CpG sites 74–78 and found that setting a higher threshold of 21% resulted in a significant increase in OS between unmethylated and methylated groups (13.3 vs. 20.4 months).^15^ A more recent Cochrane review found that, in 2 of the 3 good quality studies available, a threshold of 9% mean methylation for CpG sites 74–78 performed better than higher thresholds of 28% or 29%, though data was limited.^8^ The review found that targeting multiple CpG sites is likely to be preferable to targeting a single CpG site, and reported that cutoff thresholds varied from 4% to 25% in studies assessing single CpG sites, and from 2.68% to 35% in those assessing multiple CpG sites.^8^

In addition to the binary methylated vs unmethylated categorization, some studies have reported a gray zone of intermediate values, which can be considered a group of borderline methylated or low-level methylated samples.^13,16–20^ Recent EANO guidance advises that a gray zone must be clearly communicated and combined with other histological and pathological information for clinical decisions to be made.^6^ The gray zone could be of particular importance when considering treatment options for elderly patients, since it has been shown that temozolomide chemotherapy can be detrimental compared to radiotherapy, for patients over the age of 65 with an unmethylated MGMT status.^21,22^ Hegi et al comment the gray zone could distinguish those who are “truly” unmethylated, to ensure only these patients are enrolled in the test arm of clinical trials where temozolomide is omitted.^13^ In a recent clinical trial analysis, Hegi et al found that glioblastoma with truly unmethylated MGMT in the elderly patient population aged over 60, derived no benefit from TMZ treatment.^23^

The gray zone threshold is therefore of significant clinical importance. In their validation study, Quillien et al compared pyrosequencing and semi-quantitative methylation-specific PCR assays, using both frozen and formalin-fixed and paraffin-embedded (FFPE) samples.^16^ The study found MGMT status can reliably be investigated in local laboratories and recommended the categorization unmethylated ≤8% (median OS 15.9 months), gray zone 9%–12% (21.7 months), methylated ≥13% (35.6 months) for CpG sites 74–78, based on survivals and concordance between frozen and FFPE samples. The study observed discordant results between FFPE and frozen samples in the gray zone, where progression-free survival was no better in the gray zone than the unmethylated group in FFPE samples.

In contrast, the recent study by Hosoya et al aimed to determine the optimal methylation cutoff value for newly diagnosed GBM patients with residual tumors following surgery, who were treated with radiotherapy and temozolomide.^17^ The study focused on the tumor volume ratio (TVR), which they defined as the tumor volume at 6 months after the initial chemoradiotherapy administration divided by the tumor volume before the start of chemoradiotherapy, measured by MRI calculations. The authors analyzed the correlation between pyrosequencing average methylation (PyroMark Q96 software) of CpG sites 74–89, with TVR and change in Karnofsky performance status. The study found that TVR decreased with an increase in MGMT promoter methylation, and recommended the categorization unmethylated ≤8.2%, gray zone 8.2%–23.9%, methylated ≥23.9%, based on ROC analysis of TVR. The study investigated survival differences within these groups and found that the gray zone had a longer OS than the unmethylated group (HR 0.36, 95% CI: 0.12–0.93, *P* = .0354), but did not find a statistically significant increase in OS from the gray zone to the methylated group (HR 0.73, 95% CI: 0.24–2.46, *P* = .3023).

We aimed to develop a translatable survival-led method to derive the optimal gray zone from MGMT pyrosequencing data.

## Materials and Methods

### Case Selection in Development Cohort

The cohort we developed our method from (the “development cohort”) consists of IDH-wild-type GBM tumor samples from adult patients diagnosed between April 2016 and August 2022 at hospitals belonging to the North Bristol NHS Foundation Trust (NBT) and University Hospitals Bristol and Weston NHS Foundation Trust (UHBW), treated according to Stupp protocol. Tumor tissues with >30% neoplastic cell content were included, as per Genomics England guidelines for solid tumors.^24^

The study was approved by the South West Research Ethics Committee, as part of identifying and validating molecular targets in brain tumor and central nervous system tissue (IRAS project ID 264520, IRAS 19/SW/0194). Human brain tissue specimens were obtained with informed consent, as requested by the local ethics committee.

### DNA Extraction and MGMT Promoter Methylation Percentages

In our development cohort, DNA was extracted and MGMT (NM_002412.5) promoter CpG island methylation status was assessed using pyrosequencing with primers, as previously described by Dunn et al.^11^ Tumor tissues were FFPE. DNA was extracted and a bisulfite conversion was performed. Repeat PCR analyses were performed on each sample, and pyrosequencing detected MGMT promoter methylation across CpG sites 72 to 83 (sites numbered according to Malley et al, 2011).^25^ Pyrosequencing is a sequence-based technology that quantifies each added nucleotide to give the percentage methylation at each CpG site assessed. Target DNA was amplified using PyroMark PCR kit (Qiagen, Cat no 978703) and pyrosequencing was undertaken using a PyroMark Q48 Autoprep (Qiagen) analyzer.

### Statistics

**Figure 1.**
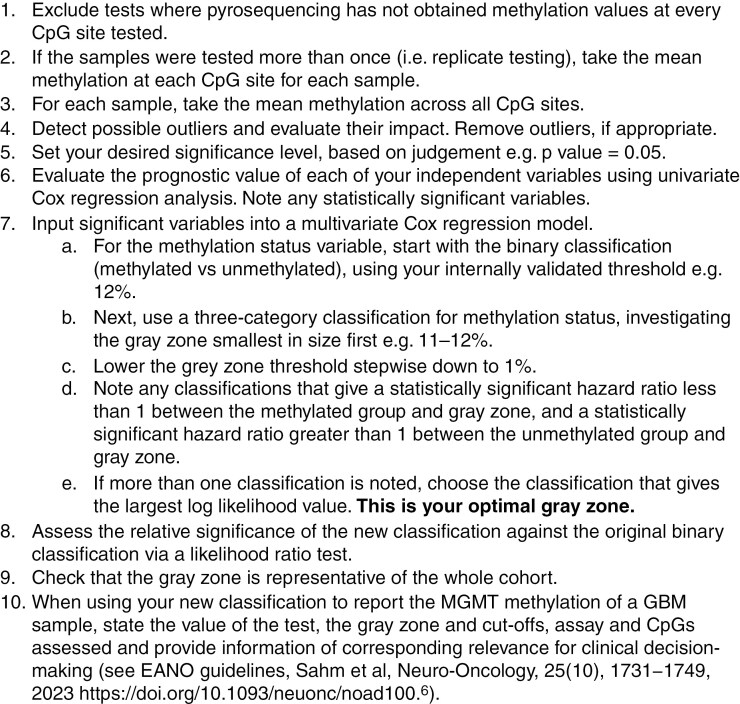
Stepwise instructions to find the optimal gray zone from pyrosequencing data. Use the R code in Supplementary Material to implement this method.

Stepwise instructions to find the optimal gray zone from pyrosequencing MGMT data can be found in Figure 1. The R code in Supplementary Material explains each step of the method clearly and has been designed to be easily applied to a new cohort.

Following internal protocol, pyrosequencing was run twice on each tumor sample for all 12 CpG sites, and any test result where pyrosequencing had failed to obtain the methylation percentage at one or more CpG sites was excluded. The mean methylation at each CpG site for each sample was calculated, before calculating the average methylation for the sample. The distribution of methylations at each of the 12 CpG sites was investigated.

Multivariate outlier detection and removal were considered. Each sample was compared against the average distribution of the 12 CpG results across the development cohort, by calculating Mahalanobis distances. The 44 samples detected did not appear to be true outliers in the context of the study and so were not removed from the study (see *Results*). However, the analysis was rerun on the development cohort excluding these potentially influential tumor samples, to evaluate their potential impact on the study.

In our development cohort, a threshold of 12% average methylation classified a sample as methylated, derived from previous intra-laboratory technical validation and a quality-controlled setup. This involved finding the integer threshold that most effectively split the cohort into 50% methylated cases and 50% unmethylated cases. This is in keeping with results from previous qMSP studies, where thresholds were defined as the value within the cohort where the probability of being methylated or unmethylated was 50%.^10,12^ The 12% average methylation threshold divided the development cohort into 49% methylated cases and 51% unmethylated cases.

Univariate Cox regression analysis investigated the prognostic value of sex, age, and average methylation. Age and methylation were investigated as both continuous variables and as categorical variables (under 65 years old/over 65 years old; methylated/gray zone/unmethylated) for application to clinical decisions. Using the 12% threshold for methylation, 11 variations of the methylated/gray zone/unmethylated classification were investigated by changing the lower threshold of the gray zone to take all integer percentages between 1% and 11%, and the Kaplan–Meier estimator was used to estimate the survival function. Note, that this stepwise method relies on having an existing threshold that classifies a sample as methylated and hence is appropriate for laboratories that fulfill this criterion.

Multivariate Cox regression models used combinations of the age, sex, and methylation variables investigated in the univariate analyses. 11 likelihood ratio test statistics were calculated between each of the 11 variations of the methylated/gray zone/unmethylated classifications and the model using the binary classification methylated/unmethylated and the result was compared against the critical value of the chi-squared distribution with one degree of freedom. The optimal gray zone was defined from the model that gave a statistically significant hazard ratio (HR) <1 between the methylated group and gray zone, and a statistically significant HR >1 between the unmethylated group and gray zone. If more than one option of the gray zone was found to fulfill these criteria, the option with the largest statistically significant log-likelihood ratio is recommended.

Distributions of age, sex, and year of diagnosis in the gray zone of the recommended model were compared with that of the overall development cohort, to check that these distributions were not skewing the results.

Throughout the analysis, a *P*-value <.05 was set for statistical significance. Multivariate outlier analysis was completed in Python version 3.11.4. All other analyses were completed in R version 4.3.1. Code can be found in Supplementary Material and ran on MGMT methylation pyrosequencing data.

### Validation Cohort

The validation cohort consists of IDH-wild-type GBM tumor samples from patients diagnosed between March 2022 and February 2024 at hospitals belonging to NHS Greater Glasgow and Clyde (NHSGGC), that were treated or had commenced treatment according to Stupp protocol. All specimens were macrodissected prior to DNA extraction to enrich tumor cells and remove contaminating non-tumor cells.

DNA was extracted and pyrosequencing was performed as previously described by Dunn et al.^11^ Tumor tissues were FFPE. DNA was extracted and a bisulfite conversion was performed. Repeat PCR analyses were performed on samples, and pyrosequencing detected MGMT promoter methylation across CpG sites 74–79 (sites numbered according to Malley et al. 2011).^25^ Target DNA was amplified using PyroMark PCR kit (Qiagen, Cat no 978703) and pyrosequencing was undertaken using a PyroMark Q48 Autoprep (Qiagen) analyzer.

In the validation cohort, 45% had been classified as methylated using a threshold of 10% average methylation derived from previous intra-laboratory technical validation and a quality-controlled setup. The previous intra-laboratory technical validation involved concordance assessment between Lothian pyrosequencing results and MS-PCR audit data, for methylation cutoffs of 8%, 9%, and 10% at CpG sites 74–79. 100% concordance (50/50 samples) was observed for the pyrosequencing methylation results obtained in the validation for all patient specimens tested from the MS-PCR audit cohort using a cutoff of 8% methylation. Of 95% concordance (19/20 samples) was observed between the pyrosequencing methylation results obtained in the validation compared to the previously reported patient specimens that were tested in Lothian using a cutoff of 8%. Intra- and inter-run repeatability showed that a specimen produced results of 8.7%, 9.2%, and 7.8% (average 8.6%, STDEV ± 0.7). This sample also underwent pyrosequencing analysis in Lothian and the result was low level methylation at 6.65%. Based on this observation, 10% was chosen as the methylation cutoff value, and pyrosequencing was repeated on tumor samples that reported 8-10% methylation. The mean methylation across the 6 CpG sites was calculated per sample.

The method proposed in this paper that was derived from the development cohort was applied to the validation cohort, using the R code in Supplementary Material and following the stepwise instructions in Figure 1.

## Results

### Cases in Development Cohort

Between April 2016 and August 2022, 733 pyrosequencing tests were conducted on GBM samples from patients at NBT and UHBW trusts. There were 33 tests that had one or more CpG sites where pyrosequencing had failed to obtain the methylation percentage, which were excluded from the study. There were 34 tests from IDH-mutant tumor samples, which were excluded to align with the WHO classification of GBM that the tumor must be wild type for IDH.^7^ The remaining 666 tests on 308 samples from 303 patients were included in the study. The clinical and demographical features of the patients are shown in Table 1.

**Table 1.** Clinical and Demographical Data of Patients

	Development cohort	Validation cohort
*Age*		
Median (range; *years)* <65 years old *(n)* ≥65 years old *(n)*	57.5 (20, 84) 219 84	61 (18, 85) 71 44
*Sex*		
Male	191	67
Female	107	48
Unknown	5	
*NHS trust*		
North Bristol NHS Foundation Trust (NBT)	290	
University Hospitals Bristol and Weston NHS Foundation Trust (UHBW)	13	
NHS Greater Glasgow and Clyde (NHSGGC)		
*Alive: dead at study*	114:189*	115 69:46**
Overall survival***		
Median [95% CI] *(months)*	10.5 [9.0, 11.2]	6.9 [5.1, 9.8]

*Measured August 04, 2023 ** Measured July 11, 2024 ***Calculated from date of diagnosis.

### MGMT Promoter Methylation

Average MGMT promoter methylation ranged from 0% to 79.5%, with an average value of 20.82 ± 21.26 (mean ± SD) %. There were 152 out of the 308 samples that met the 12% methylation threshold to be classed as methylated.

**Figure 2.**
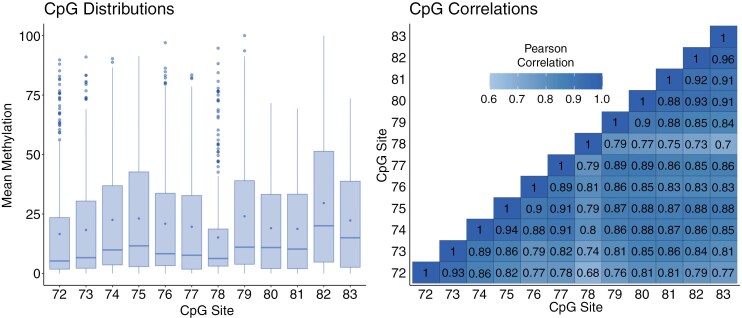
Boxplot of methylation averages at each CpG site (left) and CpG correlation heatmap (right), within the development cohort. Key: line—median, dot within interquartile range—mean.

Figure 2 shows the methylation distributions and strong correlations at each CpG. The CpG methylation distributions were similar across different age groups.

### Survivals

At the time of this study, 189 patients had died (measured on August 4, 2023). The average time to follow-up for patients alive at the time of this study was 30.31 (95% CI [26.72, 33.90]) months. The median OS was 10.55 ([9.0, 11.2]) months, calculated from the date of diagnosis to death.

#### Current classification —

**Figure 3.**
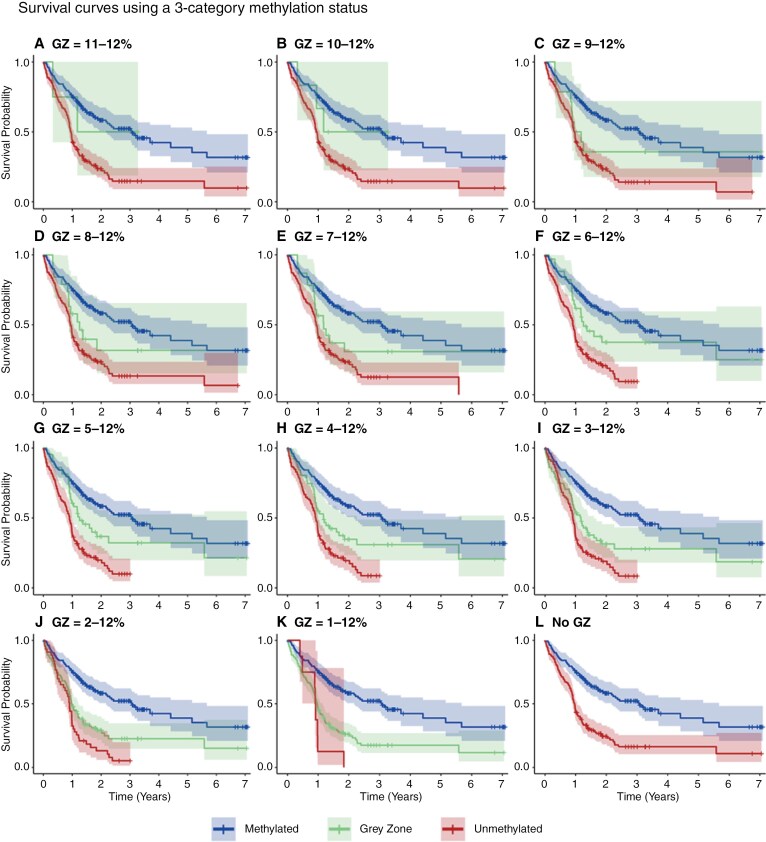
Kaplan–Meier survival plots using 12 different possible categorisations of methylation status, within the development cohort. Subplot L shows the survival curves using the binary methylation status (methylated ≥12% vs. unmethylated <12%). GZ, Gray Zone.

Using a binary definition for methylation status (methylated ≥12% vs. unmethylated <12%) showed the unmethylated group had 2.55 times the mortality risk of the methylated group (HR: 2.55, 95% CI [1.90, 3.44], *P* = 7.06 × 10^-10^). Figure 3l shows the corresponding Kaplan–Meier curves.

#### Univariate Cox proportional hazards analysis —

Univariate Cox regression analysis showed that sex had a significant relationship with OS (Male: Female HR 1.3837 [1.0145, 1.887], *P* = .0403]). Age had a significant relationship with OS when investigated as a continuous variable (HR 1.048 [1.036, 1.06], *P* = 6 × 10^-15^) and when using a threshold of 65 years (Over 65s: Under 65s HR 2.5138 [1.863, 3.392], *P* = 1.64 × 10^-9^).

Higher mean methylation was associated with better overall survival (HR 0.9807 [0.9727, 0.9887], *P* = 2.56 × 10^-6^).

Using a 3-category classification (methylated, gray zone, unmethylated) and changing the lower threshold of the gray zone between values of 11% and 1% showed that lower gray zone thresholds of 2%, 3%, 4%, and 5% gave statistically significant differences in OS between both the gray zone and methylated group and between the gray zone and unmethylated group. The HRs and *P* values can be found in Table S1. Figure 3 shows the survival plots of each possible methylation categorization, and Table 2 shows the number of patients in each category.

**Table 2.** Number of Patients and Median Survival of Each Possible Methylation Categorization, Within the Development Cohort

Methylation status categorization	Patients (*n*)	Median OS [95% CI] (months)
Methylated (≥12%)	152	11.7 [8.8, 14.7]
Gray zone (11%–12%)	4	9.0 [−inf, inf]
Unmethylated (<11%)	152	10.1 [7.6, 10.9]
Methylated (≥12%)	152	11.7 [8.8, 14.7]
Gray zone (10%–12%)	6	11.4 [−inf, inf]
Unmethylated (<10%)	150	10.1 [7.6, 10.8]
Methylated (≥12%)	152	11.7 [8.8, 14.7]
Gray zone (9%–12%)	14	10.5 [4, 14.1]
Unmethylated (<9%)	142	9.8 [7.3, 11.0]
Methylated (≥12%)	152	11.7 [8.8, 14.7]
Gray zone (8%–12%)	19	10.6 [4.3, 15.1]
Unmethylated (<8%)	137	9.75 [7.1, 10.9]
Methylated (≥12%)	152	11.7 [8.8, 14.7]
Gray zone (7%–12%)	23	10.7 [7.6, 14.1]
Unmethylated (<7%)	133	9.7 [6.5, 11.0]
Methylated (≥12%)	152	11.7 [8.8, 14.7]
Gray zone (6%–12%)	34	10.8 [7.8, 14.4]
Unmethylated (<6%)	122	9.5 [6.4, 10.8]
Methylated (≥12%)	152	11.7 [8.8, 14.7]
Gray zone (5%–12%)	43	11.0 [10.1, 14.1]
Unmethylated (<5%)	113	9.16 [6.2, 10.8]
Methylated (≥12%)	152	11.7 [8.8, 14.7]
Gray zone (4%–12%)	51	10.6 [7.3, 12.6]
Unmethylated (<4%)	105	9.7 [6.4, 11.0]
Methylated (≥12%)	152	11.7 [8.8, 14.7]
Gray zone (3%–12%)	71	10.3 [5.0, 11.8]
Unmethylated (<3%)	85	10.0 [7.1, 11.1]
Methylated (≥12%)	152	11.7 [8.8, 14.7]
Gray zone (2%–12%)	113	9.7 [6.4, 11.1]
Unmethylated (<2%)	43	10.6 [6.5, 11.8]
Methylated (≥12%)	152	11.7 [8.8, 14.7]
Gray zone (1%–12%)	148	9.8 [7.3, 10.8]
Unmethylated (<1%)	8	10.9 [4.9, 22.1]
Methylated (≥12%)	152	11.7 [8.8, 14.7]
Unmethylated (<12%)	156	10.1 [7.6, 10.9]

#### Multivariate Cox Proportional Hazards analysis —

Sex was not significant in the multivariate model that included sex, age, and mean methylation (*P* = .45). The multivariate model using age and mean methylation showed both had significant prognostic value (age: HR 1.050 [1.037, 1.062], *P* = 1.53 × 10^-15^; Mean methylation: 0.980 [0.973, 0.988], *P* = 6.02 × 10^-7^).

The results obtained using age as a binary variable (under 65 years vs. older 65 years) and methylation status as a 3-category definition (methylation ≥12%, gray zone, unmethylated) are shown in Table 3. The lower threshold for the gray zone was changed between 11% and 1% and compared against the absence of a gray zone.

**Table 3.** Hazard Ratio (HR) and *P* Value Results From 12 Multivariate Cox Proportional Hazards Models Fitted on Age and 12 Varying Definitions of Methylation Status, Within the Development Cohort

Gray zone	Over 65 years:under 65 years			Methylated:gray zone			Unmethylated:gray zone			Likelihood		Log likelihood ratio	
	HR	95% CI	*P* value	HR	95% CI	*P* value	HR	95% CI	*P* value	Score	*P* value	Test statistic	*P* value
11%–12%	2.82	[2.08, 3.83]	$2.26\times 10^{-11}$ ***	0.733	[0.178, 3.01]	.667	2.12	[0.519, 8.652]	.295	82.346	$9.63\;\times 10^{-18}$ ***	0.0344	.236
10%–12%	2.81	[2.07, 3.81]	$2.83\;\times 10^{-11}$ ***	0.644	[0.201, 2.06]	.457	1.86	[0.588, 5.91]	.290	82.309	$9.81\times 10^{-18}$ ***	0.0335	.243
9%–12%	2.82	[2.08, 3.83]	$2.36\;\times 10^{-11}$ ***	0.470	[0.234, 0.948]	.035*	1.37	[0.691, 2.723]	.366	81.833	$1.24\times 10^{-17}$ ***	0.0219	.346
8%–12%	2.80	[2.06, 3.79]	$3.6\;\times 10^{-11}$ ***	0.500	[0.269, 0.928]	.0280*	1.49	[0.814, 2.72]	.196	82.792	$7.73\times 10^{-18}$ ***	0.0452	.174
7%–12%	2.78	[2.05, 3.77]	$5.5\;\times 10^{-11}$ ***	0.492	[0.279, 0.866]	.014*	1.48	[0.85, 2.58]	.163	83.077	$6.71\times 10^{-18}$ ***	0.0520	.144
6%–12%	2.67	[1.97, 3.63]	$3.01\;\times 10^{-10}$ ***	0.548	[0.334, 0.899]	.0173*	1.77	[1.09, 2.88]	.0211*	86.855	$1.04\times 10^{-18}$ ***	0.141	.0150
5%–12%	2.75	[2.03, 3.73]	$7.3\;\times 10^{-11}$ ***	0.531	[0.342, 0.826]	.00499 **	1.81	[1.18, 2.79]	.0071**	88.894	$3.79\times 10^{-19}$ ***	0.187	.0048**
4%–12%	2.76	[2.03, 3.74]	$6.6\;\times 10^{-11}$ ***	0.479	[0.317, 0.724]	.000483 ***	1.60	[1.07, 2.40]	.023*	86.446	$1.27\times 10^{-18}$ ***	0.132	.0190*
3%–12%	2.80	[2.06, 3.79]	$3.6\;\times 10^{-11}$ ***	0.435	[0.30, 0.630]	$1.1\times 10^{-5}$ ***	1.47	[1.02, 2.14]	.0399*	85.250	$2.29\times 10^{-18}$ ***	0.104	.0380*
2%–12%	2.84	[2.10, 3.85]	$1.78\;\times 10^{-11}$ ***	0.395	[0.285, 0.547]	$2.2\;\times 10^{-8}$ ***	1.50	[1.02, 2.19]	.0388*	85.009	$2.58\times 10^{-18}$ ***	0.0980	.0438*
1%–12%	2.84	[2.09, 3.85]	$1.89\;\times 10^{-11}$ ***	0.359	[0.265, 0.488]	$4.4\;\times 10^{-11}$ ***	1.37	[0.663, 2.82]	.397	81.600	$1.39\times 10^{-17}$ ***	0.0162	.418
0%–12% (no gray zone)	2.86	[2.11, 3.88]	$1.17\;\times 10^{-11}$ ***	—	—	—	2.83	[2.097, 3.830]	$1.2\;\times 10^{-11}$ ***	80.943	$2.65\times 10^{-18}$ ***	—	—

Signif codes: *.05, **.01, ***.001.

The categorizations using gray zones 6%–12%, 5%–12%, 4%–12%, 3%–12%, and 2%–12% gave statistically significant results when comparing the OS between the gray zone & unmethylated group, and between the gray zone and methylated group. Out of these categorizations, the model using the categorization methylated ≥12%, gray zone 5%–12%, and unmethylated <5% gave the largest significant log-likelihood ratio test statistic (test statistic = 0.187, *P* = .00481). This suggests the greatest improvement from the model using the binary categorization methylated ≥12%, unmethylated <12%, and that the multivariate model using age and the new categorization was significantly better at predicting OS than the equivalent model using age and the previous binary methylation status.

The categorization methylation ≥12%, gray zone 5%–12%, and unmethylated <5% was therefore our recommended classification of methylation status for the development cohort. Adjusting for sex did not change the overall result (over 65 years: under 65 years HR 2.73 [2.01, 3.71], *P* = 1.3 × 10^−10^; methylated: gray zone HR 0.532 [0.342, 0.828], *P* = .0052; unmethylated: gray zone HR 1.77 [1.15, 2.73], *P* = .00995; male: female HR 1.16 [0.846, 1.591], *P* = .356).

The patients in the gray zone (5%–12% methylation) were representative of the development cohort regarding distributions of the year of diagnosis, age, and sex.

#### Validity of results and outlier analysis —

For the 666 pyrosequencing tests included in the development cohort, the average standard deviation for the mean methylation across the 12 CpG sites performed on each sample was 1.38%. Below the 12% methylation threshold, the average standard deviation for the mean methylation across the 12 CpG sites performed on each sample was 0.81% and no clear relationship was found between the mean methylation and degree of variation within samples.

There were 8 out of 308 samples that reported discordant methylation results when the repeat pyrosequencing was performed. An additional 7 samples would be considered discordant in the new categorization with a gray zone of 5%–12%.

A Mahalanobis distance algorithm identified samples whose distribution of the 12 CpG site methylations did not fit the general pattern within the whole development cohort, which were investigated as potential outliers. Moreover, the potential outliers included samples with contrasting methylations between those CpG sites which generally had very high Pearson correlations when considering the whole cohort, as demonstrated by Figure 2.

The 44 samples detected as potential outliers were decided to not be true outliers and were included in the study; however, the analysis was rerun excluding the 44 samples to evaluate their impact. With the 44 samples excluded, the only multivariate model that gave 3 statistically distinct survival functions was the model using age and the methylation categorization methylated ≥12%, gray zone 5%–12%, and unmethylated <5%. There were no noteworthy differences in the results of the analysis.

### Validation Cohort

The validation cohort consisted of 115 patients diagnosed between March 2022 and February 2024. The clinical and demographical features of the patients are shown in Table 1.

The following comparisons can be made between the validation cohort and the development cohort, respectively:

- Sample size (*n*): 115 versus 308.
- Proportion of patients in the respective cohort who had died at the time of study: 40% versus 62%.
- Average time to follow up (months): 16.2 versus 30.3.
- Median survival (months): 6.9 versus 10.5.

Only 9 patients in the validation cohort had an average MGMT methylation between 5% and 10%, of which only 5 had died at the time of study.

The proposed method described in Figure 1 did not identify an optimal gray zone in the validation cohort. Using a 3-category classification (methylated, gray zone, unmethylated) and changing the lower threshold of the gray zone between values of 1% and 9% did not find a statistically significant difference in OS between the gray zone and methylated group, and between the gray zone and unmethylated group.

CpG correlations, CpG distributions, Kaplan–Meier survival plots, and HRs from the 10 multivariate Cox proportional hazards models developed on the validation cohort can be found in Supplementary Material.

## Discussion

To the best of our knowledge, this is the first study that provides a method to derive the gray zone from MGMT pyrosequencing data.

### MGMT Promoter Methylation

MGMT promoter methylation was established using the pyrosequencing assay, which is currently one of the most commonly used assays for MGMT promoter methylation alongside qMSP; however, these assays may not remain the methods of choice in the future.^6^ Moreover, newer BeadChip-based arrays may become preferential due to offering genome-wide coverage, although a recent Cochrane review found that there are only a few suggestions that BeadChip-based arrays outperform MSP or pyrosequencing.^8^ A limitation of this study is that the proposed method can only be used on pyrosequencing data, as other assays generate results of different datatypes.

Tumor tissues with >30% neoplastic cell content were included in the development cohort, due to internal protocol within NBT and UHBW NHS trusts. It is therefore possible that the MGMT methylation values for the samples are a combination of various extents of tumor cell contents from the dissection. This may result in average methylation values that are not representative of tissue only containing tumor cells. This is an important limitation of the study. It may not be possible or realistic to perform tumor cell dissection in every tumor sample. Future work could investigate the impact of tumor purity by considering different cutoffs of percentage neoplastic cell content, or driver mutation VAF data. It could be of particular interest to investigate the relationship between tumor purity and the number of discordant results.

Quillien et al and Buyuktepe et al investigated the prognostic value of individual CpG methylations and suggested that methylation of particular CpG sites might predict clinical outcomes more precisely.^4,26^ Moreover, Quillien et al determined different cutoff values for different CpG sites using ROC analysis and a Cox model (4% cutoff for CpG 74, 11% cutoff for CpG 75, 4% for CpG 76, 6% for CpG 77, and 5% for CpG 78). When considering each of the 5 CpG sites separately, they found that patients who met the methylation cutoff had a longer OS, whatever the CpG position (*P* = 2.9 × 10^−4^ for CpG 74, *P* = 2.4 × 10^−5^ for CpG 75, *P* = 1.2 × 10^−4^ for CpG 76, *P* = 1.4 × 10^−5^ for CpG 77 and *P* = 3.4 × 10^−4^ for CpG 78). Buyuktepe et al investigated whether any of the CpG groups CpG 70–78, CpG 79–83, CpG 84–87, or CpG 70–87 were more prognostic than others, in 95 high-grade glioma patients. They found that patients with >30% average methylation of sites CpG 79–83 and 84–87 were statistically associated with better survival outcomes, compared to CpG 70–78 and CpG 70–87 (CpG 79–83 median OS: 0%–10% methylation 12.73 months, 10%–30% methylation 11.67 months, >30% methylation 25.80 months, *P* = .008. CpG 84–87 median OS: 0%–10% methylation 12.73 months, 10%–30% methylation 11.67 months, >30% methylation 25.80 months, *P* = .014).

We identified that using the average methylation across all CpGs tested rather than values from individual CpG sites (which may vary between laboratories) gave a methodology that is reproducible. This is in keeping with the Cochrane meta-analysis by McAleenan et al, which did not provide strong evidence about the best CpG sites or threshold.^8^ In our study, the average standard deviation for the replicate tests performed on each sample was 1.38%, raising the possibility that different centers could derive different optimal gray zones when testing the same CpG sites. Recent EANO guidelines suggest that the establishment of an MGMT assay for clinical application requires intra-laboratory technical validation.^6^

### Gray Zone Methodology

In our study, the 12% methylation threshold gave a statistically significant difference in OS, where the unmethylated group had 2.55 times the mortality risk of the methylated group (HR: 2.55, 95% CI [1.90, 3.44], *P* = 7.06 × 10^-10^).

#### Univariate Cox proportional hazards analysis —

The first step in our gray zone methodology was to establish the prognostic value of each variable (age, sex, and average methylation) using a univariate Cox proportional hazards model. In our development cohort, univariate analysis showed that age, sex, and average methylation were significantly related to OS. This is in keeping with existing literature, where age is widely accepted as a key factor in the survival of gliomas, and females have been found to have a statistically significant survival advantage compared to males.^27–29^ Due to data accessibility and lack of data objectivity, this methodology did not incorporate factors such as tumor location, extent of resection, or comorbidities.

#### Multivariate Cox Proportional Hazards analysis —

The second step in our gray zone methodology was to evaluate the collective prognostic value of the significant variables (age, sex, and average methylation) using a multivariate Cox proportional hazards model. Age, sex, and methylation were investigated as categorical variables due to their direct application to clinical decisioning.

Our methodology identified an optimal categorization of methylated ≥12%, gray zone 5%–12%, and unmethylated <5%, within the development cohort of 308 GBM samples. Once adjusted for age, the methylated group had a 47% decreased mortality risk to the gray zone, and the unmethylated group had an 81% increased mortality risk to the gray zone. Using the new categorization, we identified 43 of the 156 patients who were previously considered unmethylated to now be considered in the gray zone. Hegi et al. found that the gray zone may confer some sensitivity to temozolomide treatment, and hence, our gray zone identified over a quarter of the unmethylated group that could potentially benefit from temozolomide.^13^

Other studies that report a gray zone in pyrosequencing data include Quillien et al., who similarly used a 12% methylation threshold but found a narrower margin for the gray zone (9%–12%).^16^ The study considered both OS and concordance between FFPE and frozen samples when deriving the thresholds, and did not observe a clear benefit of temozolomide in the gray zone group. Hosoya et al used a much higher methylation threshold of 23.9% and found a wider margin for the gray zone (8.2%–23.9%).^17^ The study derived the thresholds based on the change in tumor volume as opposed to OS and did not observe a statistically significant difference in OS between the gray zone and methylated group.

Our proposed methodology found a statistically significant difference in survival between both the gray zone and unmethylated group and the gray zone and methylated group, for the first time from pyrosequencing data. This method design ensures that there is reason to move from a binary classification (unmethylated vs. methylated) to a 3-category classification (unmethylated vs. gray zone vs. methylated) due to the impact on choices of treatment and survivals. Moreover, one could argue to change the methylation threshold within a binary classification, if a statistically significant difference was not observed between both the gray zone and unmethylated group and the gray zone and methylated group.

A limitation of our proposed method is that an adequate proportion of patients in the cohort are required to have average methylations that fall in the intermediate range, in order to power the study. It is likely that both the small sample size and the short time to follow-up contributed to the lack of statistically significant findings in the validation cohort. We advise research centers to carefully consider the cohort size and time to follow up before using our methodology.

In conclusion, we have developed a translatable method which can be used to identify the gray zone from intra-laboratory pyrosequencing data, in keeping with the recent EANO guidelines.^6^ In the development cohort, our method reports the first gray zone with a statistically different survival to that of both the methylated and unmethylated groups, from pyrosequencing data.

## Supplementary Material

vdaf061_suppl_Supplementary_Material

## Data Availability

The data will be made available upon reasonable request by contacting Polly Taylor, us22212@bristol.ac.uk.
