# Supplementary material for: Defining the recommended gray zone in O6-methylguanine-DNA methyltransferase promoter methylation pyrosequencing reporting: A robust, translatable method to implement new EANO guidelines
Source: Neurooncol Adv. 2025 Mar 22;7(1):vdaf061. doi: 10.1093/noajnl/vdaf061 (PMC12121717; doi:10.1093/noajnl/vdaf061)
Supplement: vdaf061_suppl_Supplementary_Material [file vdaf061_suppl_supplementary_material.docx]

**Development Cohort - Univariate Cox Proportional Hazards analysis**

Table 1 - Hazard ratio and p value results from 12 univariate Cox proportional hazards models fitted on 12 varying definitions of methylation status, within the development cohort. Signif codes: *0.05, **0.01, ***0.001.

| **Gray zone** | **Methylated:Gray zone** | | | **Unmethylated:Gray Zone** | | |
| --- | --- | --- | --- | --- | --- | --- |
|  | **HR** | **95% CI** | **p value** | **HR** | **95% CI** | **p value** |
| 11-12% | 0.987 | [0.242, 4.03] | .985 | 2.60 | [0.639, 10.55] | .182 |
| 10-12% | 0.881 | [0.277, 2.80] | .829 | 2.33 | [0.738, 7.35] | .149 |
| 9-12% | 0.592 | [0.296, 1.19] | .139 | 1.58 | [0.80, 3.13] | .187 |
| 8-12% | 0.625 | [0.339, 1.15] | .132 | 1.72 | [0.944, 3.13] | .076 |
| 7-12% | 0.620 | [0.355, 1.08] | .0929 | 1.74 | [1.01, 3.01] | .046* |
| 6-12% | 0.684 | [0.420, 1.11] | .126 | 2.13 | [1.32, 3.44] | .002** |
| 5-12% | 0.622 | [0.402, 0.963] | .0334* | 1.97 | [1.29, 3.06] | .0017** |
| 4-12% | 0.561 | [0.373, 0.845] | .00564** | 1.77 | [1.18, 2.65] | .0054** |
| 3-12% | 0.498 | [0.345, 0.719] | .000201*** | 1.58 | [1.10, 2.29] | .0145* |
| 2-12% | 0.442 | [0.320, 0.610] | $7.0 \times{10}^{-7}$  *** | 1.54 | [1.05, 2.26] | .025* |
| 1-12% | 0.401 | [0.296, 0.542] | $2.7 \times{10}^{-9}$  *** | 1.60 | [0.78, 3.29] | .20 |
| 0-12% (no gray zone) |  |  |  | 2.55 | [1.895,  3.438] | $7.06\times{10}^{-10}$  *** |

**Validation Cohort – MGMT Promoter Methylation**

*
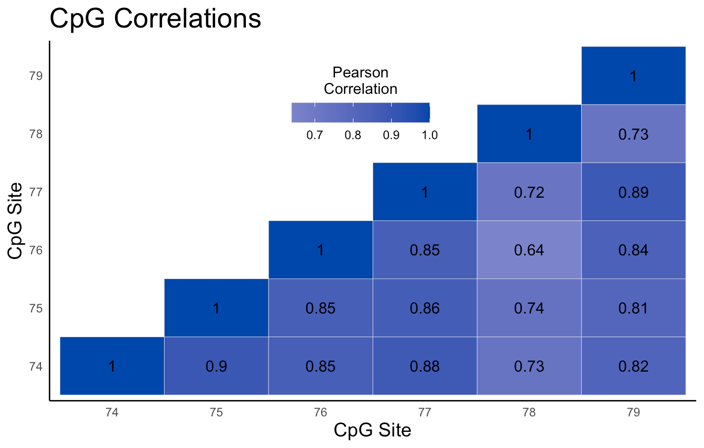

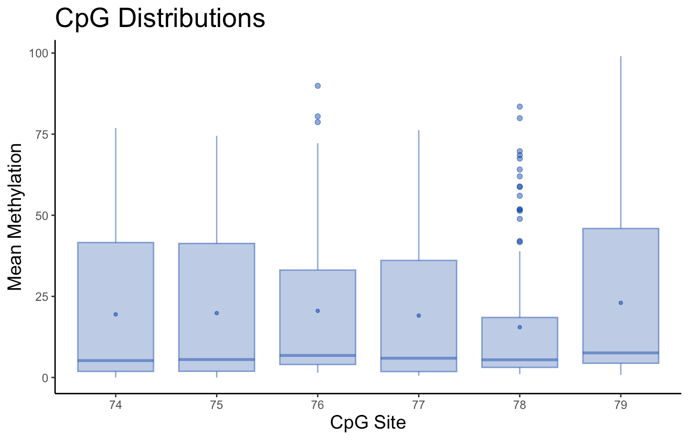
*

Figure 1 - CpG correlation heatmap (left) and boxplot of methylation averages at each CpG site (right), within the validation cohort. Key: line – median, dot within interquartile range – mean.

*
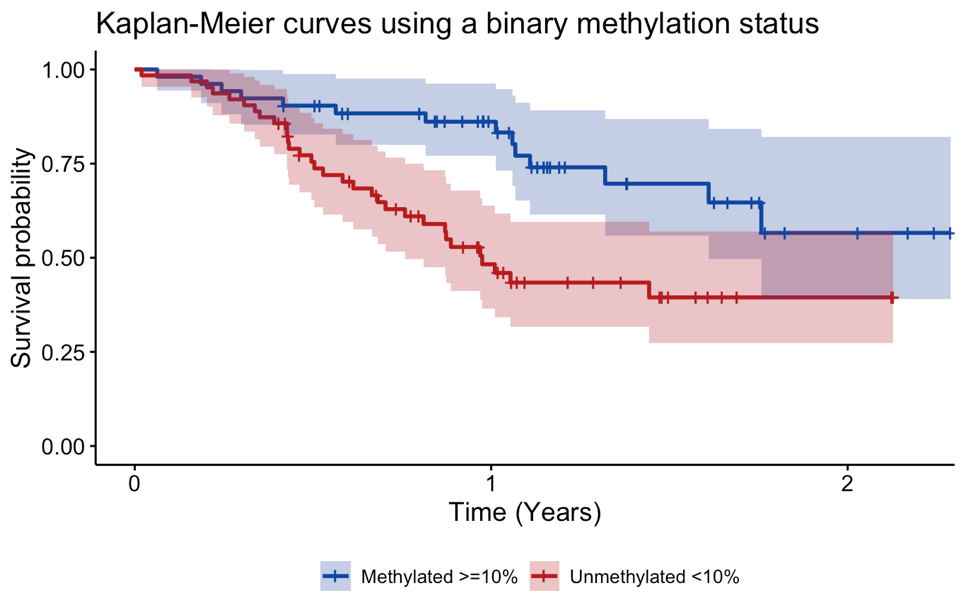
*

Figure 2 – Kaplan-Meier survival plot using the binary methylation status (methylated >=10% vs unmethylated <10%), within the validation cohort.

**Validation Cohort – Univariate Cox Proportional Hazards analysis**

*
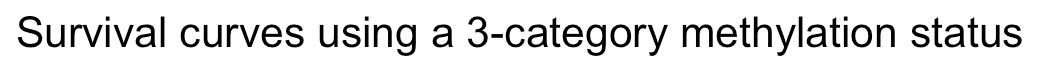
*

*
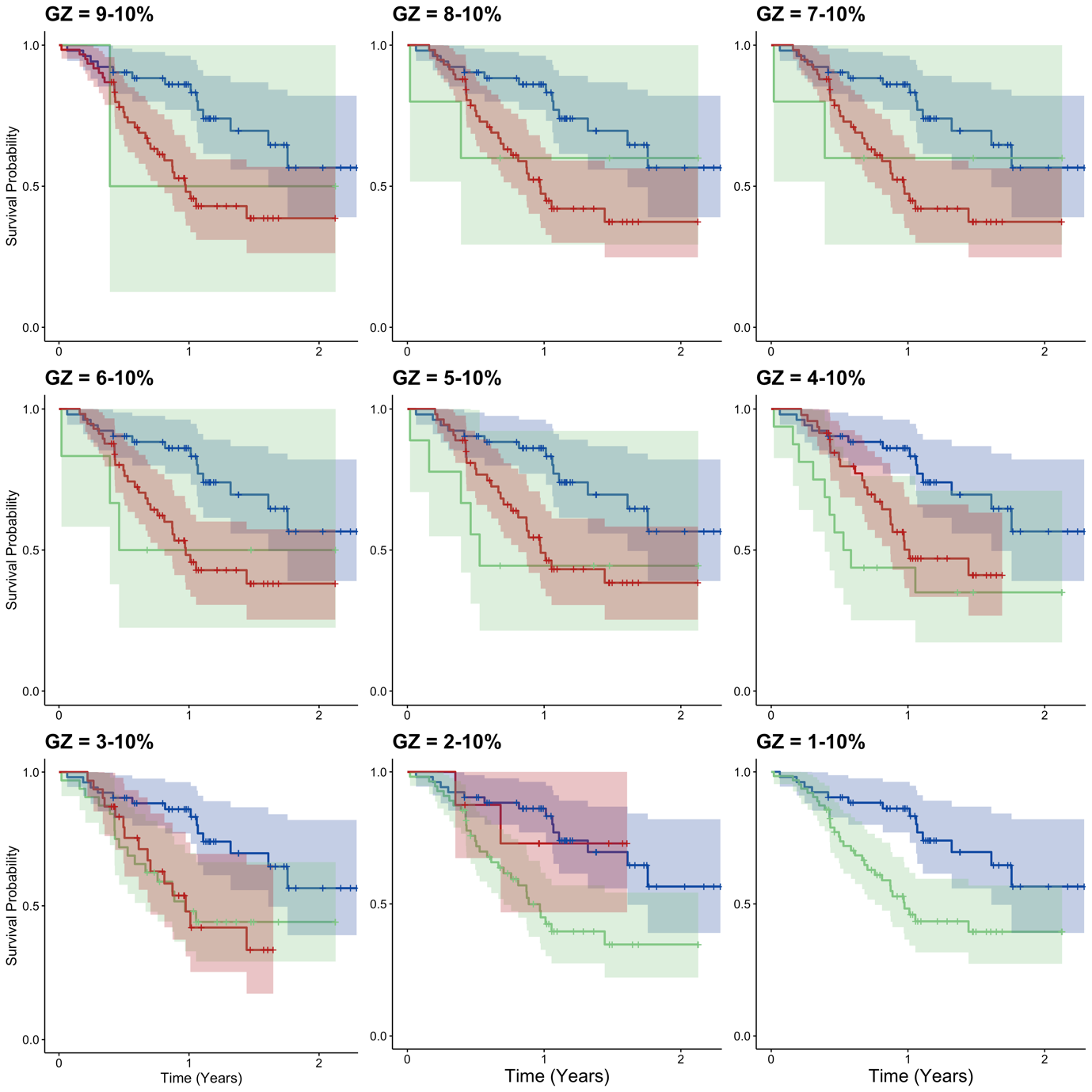
*

*
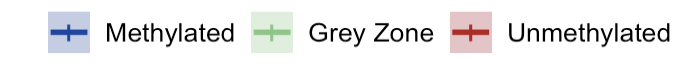
*

Figure 3 – Kaplan-Meier survival plots using 10 different possible categorisations of methylation status, within the validation cohort. GZ: Gray Zone.

**Validation Cohort – Multivariate Cox Proportional Hazards analysis**

Table 2 – Hazard ratio and p value results from 10 multivariate Cox proportional hazards models fitted on age and 10 varying definitions of methylation status, within the validation cohort. Signif codes: .0.1, *0.05, **0.01, ***0.001.

| **Gray zone** | **Over 65 years:Under 65 years** | | | **Methylated:Gray zone** | | | **Unmethylated:Gray Zone** | | |
| --- | --- | --- | --- | --- | --- | --- | --- | --- | --- |
|  | **HR** | **95% CI** | **p value** | **HR** | **95% CI** | **p value** | **HR** | **95% CI** | **p value** |
| 9-10% | 1.9988 | [1.08315, 3.689] | 0.0267* | 0.3641 | [0.04547, 2.915] | 0.3411 | 1.2136 | [0.15882, 9.274] | 0.8520 |
| 8-10% | 1.998 | [1.08532, 3.676] | 0.0262* | 0.394 | [0.08726, 1.779] | 0.2259 | 1.332 | [0.31466, 5.642] | 0.6967 |
| 7-10% | 1.998 | [1.08532, 3.676] | 0.0262* | 0.394 | [0.08726, 1.779] | 0.2259 | 1.332 | [0.31466, 5.642] | 0.6967 |
| 6-10% | 2.0174 | [1.09400, 3.720] | 0.0246* | 0.2816 | [0.07822, 1.014] | 0.0525. | 0.9262 | [0.27864, 3.079] | 0.9005 |
| 5-10% | 2.0270 | [1.09927, 3.7377] | 0.02363* | 0.2458 | [0.08564, 0.7052] | 0.00907** | 0.7871 | [0.30088, 2.0590] | 0.62561 |
| 4-10% | 2.0928 | [1.12824, 3.8822] | 0.019144* | 0.2067 | [0.08749, 0.4883] | 0.000325*** | 0.6031 | [0.28176, 1.2907] | 0.192696 |
| 3-10% | 2.0286 | [1.0917, 3.7692] | 0.02524* | 0.2935 | [0.1376, 0.6264] | 0.00153** | 0.9455 | [0.4655, 1.9204] | 0.87684 |
| 2-10% | 2.2503 | [1.20363, 4.2072] | 0.011069* | 0.2544 | [0.12744, 0.5079] | 0.000104*** | 0.3047 | [0.07157, 1.2970] | 0.107804 |
| 1-10% | 2.012 | [1.0931, 3.7024] | 0.024712* | 0.302 | [0.1545, 0.5901] | 0.000461*** | - | - | - |
| 0-10% (no gray zone) | 2.012 | [1.0931, 3.7024] | 0.024712* | - | - | - | 3.312 | [1.695, 6.472] | 0.000461*** |

**R Code**

Computed in version 4.3.1.

# ---------------------------------------------------------------------------- #

# ---------------------------- Information ----------------------------------- #

# ---------------------------------------------------------------------------- #

# -------- This code analyses MGMT methylation pyrosequencing data --------- #

# ---------------------------------------------------------------------------- #

# ---------------------------------------------------------------------------- #

# ------------------------- REQUIRED PACKAGES -------------------------------- #

# ------------------------- car v3.1.2 --------------------------------- #

# ------------------------- dplyr v1.1.2 --------------------------------- #

# ------------------------- ggplot2 v3.4.2 --------------------------------- #

# ------------------------- gridExtra v2.3 -------------------------------- #

# ------------------------- reshape2 v1.4.4 -------------------------------- #

# ------------------------- survival v3.5.5 -------------------------------- #

# ------------------------- survminer v0.4.9 --------------------------------- #

# ---------------------------------------------------------------------------- #

# ---------------------------------------------------------------------------- #

# ------------------------------- INPUT -------------------------------------- #

# ---------------------------------------------------------------------------- #

# Requires csv file with following column names, though code could be adapted: #

# #

# COLUMN NAME DESCRIPTION, DATATYPE, VALUES #

# ID: Patient ID, datatype: int #

# Sex: Patient Sex, datatype:char (values: "M" or "F") #

# Age_Diagnosis: Age of patient at diagnosis, datatype: int #

# Date_Of_Diagnosis: Date of diagnosis, datatype: date ("yyyy-mm-dd") #

# Date_Of_Death: Date of death, datatype: date ("yyyy-mm-dd", #

# value = NA if patient is alive at time of study) #

# CpG_1_Mean: Average methylation at 1st CpG site for the sample, #

# datatype: numeric #

# CpG_2_Mean: Average methylation at 2nd CpG site for the sample, #

# datatype: numeric #

# ... ... #

# CpG_12_Mean: Average methylation at 12th CpG site for the sample, #

# datatype: numeric #

# #

# ---------------------------------------------------------------------------- #

# ----- Code assumes there are no rows with any CpG methylations missing ----- #

# Code will need to be adapted if data contains more or less than 12 CpG sites #

# ---------------------------------------------------------------------------- #

# ---------------------------------------------------------------------------- #

# ------------------------------- METHOD ------------------------------------- #

# ---------------------------------------------------------------------------- #

# 1. Import data - this file works on mock data in the absence of real data -- #

# 2. Create calculated columns ----------------------------------------------- #

# 3. Exploratory Data Analysis - look at methylation distributions, ---------- #

# correlations, paired t-tests on CpG methylations, ----------------------- #

# 4. Univariate analysis - explores prognostic value of sex, age, methylation. #

# Uses log rank tests, Kaplan-Meier estimates and Cox proportional hazard - #

# models ------------------------------------------------------------------ #

# 5. Multivariate analysis- explores prognostic value of sex, age, methylation #

# Uses Cox proportional hazards models ------------------------------------ #

# ---------------------------------------------------------------------------- #

# ---------------------------------------------------------------------------- #

# ------------------------------- OUTPUT ------------------------------------- #

# ---------------------------------------------------------------------------- #

# Plots - boxplot, correlation heatmap, Kaplan-Meier for different gray zones #

# - Model output - hazard ratios and p values for different gray zone options #

# ---------------------------------------------------------------------------- #

### Set up environment and load data -------------------------------------------

## Install any packages not already installed

# install.packages("car")

## Import packages

library(car)

library(dplyr)

library(ggplot2)

library(gridExtra)

library(reshape2)

library(survival)

library(survminer)

## Import data and create dataframe

# Mock data follows, with desired format and datatypes:

df <- data.frame (ID = seq(from = 1, to = 50),

Sex = sample(c("M","F"), 50, replace = TRUE),

Age_Diagnosis = round(runif(n = 50, min = 5.0, max = 80.0), digits = 0),

Date_Of_Diagnosis = sample(seq(as.Date("2016-04-01"), as.Date("2022-08-01"), by="day"), 50),

CpG_1_Mean = round(runif(n = 50, min = 0.0, max = 20.0), digits = 1),

CpG_2_Mean = round(runif(n = 50, min = 0.0, max = 20.0), digits = 1),

CpG_3_Mean = round(runif(n = 50, min = 0.0, max = 20.0), digits = 1),

CpG_4_Mean = round(runif(n = 50, min = 0.0, max = 20.0), digits = 1),

CpG_5_Mean = round(runif(n = 50, min = 0.0, max = 20.0), digits = 1),

CpG_6_Mean = round(runif(n = 50, min = 0.0, max = 20.0), digits = 1),

CpG_7_Mean = round(runif(n = 50, min = 0.0, max = 20.0), digits = 1),

CpG_8_Mean = round(runif(n = 50, min = 0.0, max = 20.0), digits = 1),

CpG_9_Mean = round(runif(n = 50, min = 0.0, max = 20.0), digits = 1),

CpG_10_Mean = round(runif(n = 50, min = 0.0, max = 20.0), digits = 1),

CpG_11_Mean = round(runif(n = 50, min = 0.0, max = 20.0), digits = 1),

CpG_12_Mean = round(runif(n = 50, min = 0.0, max = 20.0), digits = 1)

)

df$Date_Of_Death = df$Date_Of_Diagnosis + round(runif(n = 1, min = 1, max = 1000), digits = 0)

df$Date_Of_Death[which(df$Date_Of_Death %in% sample(df$Date_Of_Death, 20))]<-NA

# Replace with real data, which should be saved as a csv titled 'Data.csv' in working directory

# df <- read.csv("Data.csv")

## Create calculated columns

df <- df %>%

mutate(

Age_65_threshold = case_when(

Age_Diagnosis >=65 ~ 1,

Age_Diagnosis <65 ~ 0) # Flag for if patient was over age of 65 at diagnosis

, Date_Of_Diagnosis=as.Date(Date_Of_Diagnosis, format = "%d/%m/%Y")

, Date_Of_Death=as.Date(Date_Of_Death, format = "%d/%m/%Y")

, Died = ifelse(is.na(Date_Of_Death), 0, 1) # Flag for if patient has died

, Survival_Days = case_when(

Died == 1 ~ as.double(difftime(Date_Of_Death,Date_Of_Diagnosis, units = c("days"))),

Died == 0 ~ as.double(difftime(Sys.Date(),Date_Of_Diagnosis, units = c("days"))) ) # Length of survival in days. Calculated as diagnosis to death for patients who have died, or diagnosis to date of study (today) for patients who are alive

# Died == 0 ~ as.double(difftime(as.Date("2023-08-04"),Date_Of_Diagnosis, units = c("days"))) ) # Length of survival in days. Keep same date of "today" for reproducing output.

, Mean_methylation = rowMeans(select(df, c(CpG_1_Mean,CpG_2_Mean,CpG_3_Mean,CpG_4_Mean,CpG_5_Mean,CpG_6_Mean,CpG_7_Mean,CpG_8_Mean,CpG_9_Mean,CpG_10_Mean,CpG_11_Mean,CpG_12_Mean))) # mean methylation across the 12 CpG sites for the sample

, Methylation_Status_No_GZ = case_when(

Mean_methylation>=12 ~ "Methylated",

Mean_methylation<12 ~ "Unmethylated") #Categorisation: Methylated >=12%, unmethylated <12%

, Methylation_Status_11 = case_when(

Mean_methylation>=12 ~ "Methylated",

between(Mean_methylation, 11, 12) ~ "Grey Zone",

Mean_methylation<11 ~ "Unmethylated") #Categorisation: Methylated >=12%, grey zone 11-12%, unmethylated <11%

, Methylation_Status_10 = case_when(

Mean_methylation>=12 ~ "Methylated",

between(Mean_methylation, 10, 12) ~ "Grey Zone",

Mean_methylation<10 ~ "Unmethylated") #Categorisation: Methylated >=12%, grey zone 10-12%, unmethylated <10%

, Methylation_Status_9 = case_when(

Mean_methylation>=12 ~ "Methylated",

between(Mean_methylation, 9, 12) ~ "Grey Zone",

Mean_methylation<9 ~ "Unmethylated") #Categorisation: Methylated >=12%, grey zone 9-12%, unmethylated <9%

, Methylation_Status_8 = case_when(

Mean_methylation>=12 ~ "Methylated",

between(Mean_methylation, 8, 12) ~ "Grey Zone",

Mean_methylation<8 ~ "Unmethylated") #Categorisation: Methylated >=12%, grey zone 8-12%, unmethylated <8%

, Methylation_Status_7 = case_when(

Mean_methylation>=12 ~ "Methylated",

between(Mean_methylation, 7, 12) ~ "Grey Zone",

Mean_methylation<7 ~ "Unmethylated") #Categorisation: Methylated >=12%, grey zone 7-12%, unmethylated <7%

, Methylation_Status_6 = case_when(

Mean_methylation>=12 ~ "Methylated",

between(Mean_methylation, 6, 12) ~ "Grey Zone",

Mean_methylation<6 ~ "Unmethylated") #Categorisation: Methylated >=12%, grey zone 6-12%, unmethylated <6%

, Methylation_Status_5 = case_when(

Mean_methylation>=12 ~ "Methylated",

between(Mean_methylation, 5, 12) ~ "Grey Zone",

Mean_methylation<5 ~ "Unmethylated") #Categorisation: Methylated >=12%, grey zone 5-12%, unmethylated <5%

, Methylation_Status_4 = case_when(

Mean_methylation>=12 ~ "Methylated",

between(Mean_methylation, 4, 12) ~ "Grey Zone",

Mean_methylation<4 ~ "Unmethylated") #Categorisation: Methylated >=12%, grey zone 4-12%, unmethylated <4%

, Methylation_Status_3 = case_when(

Mean_methylation>=12 ~ "Methylated",

between(Mean_methylation, 3, 12) ~ "Grey Zone",

Mean_methylation<3 ~ "Unmethylated") #Categorisation: Methylated >=12%, grey zone 3-12%, unmethylated <3%

, Methylation_Status_2 = case_when(

Mean_methylation>=12 ~ "Methylated",

between(Mean_methylation, 2, 12) ~ "Grey Zone",

Mean_methylation<2 ~ "Unmethylated") #Categorisation: Methylated >=12%, grey zone 2-12%, unmethylated <2%

, Methylation_Status_1 = case_when(

Mean_methylation>=12 ~ "Methylated",

between(Mean_methylation, 1, 12) ~ "Grey Zone",

Mean_methylation<1 ~ "Unmethylated") #Categorisation: Methylated >=12%, grey zone 1-12%, unmethylated <1%

)

### Exploratory Data Analysis --------------------------------------------------

## Box plot

df_bp <- melt(df, id.vars='ID', measure.vars=c('CpG_1_Mean','CpG_2_Mean','CpG_3_Mean','CpG_4_Mean','CpG_5_Mean','CpG_6_Mean','CpG_7_Mean','CpG_8_Mean','CpG_9_Mean','CpG_10_Mean','CpG_11_Mean','CpG_12_Mean'))

ggplot(df_bp) +

geom_boxplot(aes(x=variable, y=value), colour="#0047AB80", fill="#0047AB50") +

stat_summary(aes(x=variable,y=value), fun=mean, geom="point", colour="#0047AB90", shape=20, size=1.5) +

scale_x_discrete(labels=c("72","73","74","75","76","77","78","79","80","81","82","83")) +

labs(x = "CpG Site", y = "Mean Methylation", title = "CpG Distributions") +

theme(panel.background = element_blank(),

axis.line = element_line(colour = "black"),

plot.title = element_text(size = 20),

title=element_text(size=14))

## Correlation Heatmap

cormat <- round(cor(df[,c("CpG_1_Mean","CpG_2_Mean","CpG_3_Mean","CpG_4_Mean","CpG_5_Mean","CpG_6_Mean","CpG_7_Mean","CpG_8_Mean","CpG_9_Mean","CpG_10_Mean","CpG_11_Mean","CpG_12_Mean")]),2)

get_upper_tri <- function(cormat){

cormat[lower.tri(cormat)]<- NA

return(cormat)

}

upper_tri <- get_upper_tri(cormat)

melted_cormat <- melt(upper_tri, na.rm = TRUE)

ggplot(data = melted_cormat, aes(Var2, Var1, fill = value))+

geom_tile(color = "black")+

scale_fill_gradient2(high = "#0047AB", mid = "#4682B4", low="#A7C7E7",

# midpoint = 0.85,

# limit = c(0.6,1),

space = "Lab",

name="Pearson\nCorrelation") +

scale_x_discrete(labels=c("72","73","74","75","76","77","78","79","80","81","82","83")) +

scale_y_discrete(labels=c("72","73","74","75","76","77","78","79","80","81","82","83")) +

labs(x = "CpG Site", y = "CpG Site", title = "CpG Correlations") +

geom_text(aes(Var2, Var1, label = value), color = "black", size = 4) +

theme(

panel.border = element_blank(),

panel.background = element_blank(),

axis.ticks = element_blank(),

axis.line = element_line(colour = "black"),

axis.title=element_text(size=14),

legend.justification = c(1, 0),

legend.position = c(0.6, 0.7),

plot.title = element_text(size = 20),

legend.direction = "horizontal")+

guides(fill = guide_colorbar(barwidth = 7, barheight = 1, title.position = "top", title.hjust = 0.5))

## Kaplan Meier Curve - All patients

fit <- survfit(Surv(Survival_Days, Died) ~ 1, data = df)

ggsurvplot(

fit,

data = df,

xscale = 365.25, # scale x axis to show years not days

break.x.by = 365.25,

size = 1,

palette = c("#A7C7E7"),

conf.int = TRUE,

xlab = "Time (Years)",

title = 'Kaplan Meier - All patients',

legend = 'none'

)

### Univariate analysis --------------------------------------------------------

## Sex

summary(coxph(Surv(Survival_Days, Died) ~ Sex, data = df)) # Cox

## Age, as a continuous variable and also as a binary variable (under & over 65s)

summary(coxph(Surv(Survival_Days, Died) ~ Age_Diagnosis, data = df)) # continuous

summary(coxph(Surv(Survival_Days, Died) ~ Age_65_threshold, data = df)) # binary (under & over 65s)

## Mean methylation - continuous

summary(coxph(Surv(Survival_Days, Died) ~ Mean_methylation, data = df)) # A negative coef means higher methylation is associated with better survival

## Mean methylation - Binary status methylated (>=12%) vs unmethylated (<12%)

methfit_no_GZ <- survfit(Surv(Survival_Days, Died) ~ Methylation_Status_No_GZ, data = df)

ggsurvplot(

methfit_no_GZ,

data = df,

xscale = 365.25,

break.x.by = 365.25,

size = 1,

palette =c("#0D47A1", "#B71C1C"),

conf.int = TRUE,

xlab = "Time (Years)",

legend.labs = c("Methylated >=12%","Unmethylated <12%"),

title = 'Kaplan-Meier curves using a binary methylation status',

legend.title="",

legend = "bottom"

)

## Mean methylation - 3 category methylation status (11 options)

methfit11 <- survfit(Surv(Survival_Days, Died) ~ Methylation_Status_11, data = df) # GZ: 11-12%

methfit10 <- survfit(Surv(Survival_Days, Died) ~ Methylation_Status_10, data = df) # GZ: 10-12%

methfit9 <- survfit(Surv(Survival_Days, Died) ~ Methylation_Status_9, data = df) # GZ: 9-12%

methfit8 <- survfit(Surv(Survival_Days, Died) ~ Methylation_Status_8, data = df) # GZ: 8-12%

methfit7 <- survfit(Surv(Survival_Days, Died) ~ Methylation_Status_7, data = df) # GZ: 7-12%

methfit6 <- survfit(Surv(Survival_Days, Died) ~ Methylation_Status_6, data = df) # GZ: 6-12%

methfit5 <- survfit(Surv(Survival_Days, Died) ~ Methylation_Status_5, data = df) # GZ: 5-12%

methfit4 <- survfit(Surv(Survival_Days, Died) ~ Methylation_Status_4, data = df) # GZ: 4-12%

methfit3 <- survfit(Surv(Survival_Days, Died) ~ Methylation_Status_3, data = df) # GZ: 3-12%

methfit2 <- survfit(Surv(Survival_Days, Died) ~ Methylation_Status_2, data = df) # GZ: 2-12%

methfit1 <- survfit(Surv(Survival_Days, Died) ~ Methylation_Status_1, data = df) # GZ: 1-12%

## Survival Plots -------------------------------------------------------------------

# Create a list to fill with Kaplan Meier subplots of each possible categorisation

splots <- list()

# 11%

splots[[1]] <- ggsurvplot(

methfit11,

data = df,

size = 1,

xscale = 365.25,

break.x.by = 365.25,

break.y.by = 0.5,

palette = c("#81C784","#0D47A1", "#B71C1C"),

conf.int = TRUE,

title = 'GZ = 11-12%',

xlab = NULL,

ylab = 'Survival Probability',

font.title = c(20, "bold"),

font.y = c(18),

legend = "none"

)

# 10%

splots[[5]] <- ggsurvplot(

methfit10,

data = df,

size = 1,

xscale = 365.25,

break.x.by = 365.25,

break.y.by = 0.5,

palette = c("#81C784","#0D47A1", "#B71C1C"),

conf.int = TRUE,

title = 'GZ = 10-12%',

font.title = c(20, "bold"),

xlab = NULL,

ylab = NULL,

legend = "none"

)

# 9%

splots[[9]] <- ggsurvplot( # Note index in list isn't linear due to desired position on plot

methfit9,

data = df,

size = 1,

xscale = 365.25,

break.x.by = 365.25,

break.y.by = 0.5,

palette = c("#81C784","#0D47A1", "#B71C1C"),

conf.int = TRUE,

title = 'GZ = 9-12%',

font.title = c(20, "bold"),

xlab = NULL,

ylab = NULL,

legend = "none"

)

# 8%

splots[[2]] <- ggsurvplot(

methfit8,

data = df,

size = 1,

xscale = 365.25,

break.x.by = 365.25,

break.y.by = 0.5,

palette = c("#81C784","#0D47A1", "#B71C1C"),

conf.int = TRUE,

title = 'GZ = 8-12%',

font.title = c(20, "bold"),

font.y = c(18),

xlab = NULL,

ylab = 'Survival Probability',

legend = "none"

)

# 7%

splots[[6]] <- ggsurvplot(

methfit7,

data = df,

size = 1,

xscale = 365.25,

break.x.by = 365.25,

break.y.by = 0.5,

palette = c("#81C784","#0D47A1", "#B71C1C"),

conf.int = TRUE,

title = 'GZ = 7-12%',

font.title = c(20, "bold"),

xlab = NULL,

ylab = NULL,

legend = "none"

)

# 6%

splots[[10]] <- ggsurvplot(

methfit6,

data = df,

size = 1,

xscale = 365.25,

break.x.by = 365.25,

break.y.by = 0.5,

palette = c("#81C784","#0D47A1", "#B71C1C"),

conf.int = TRUE,

title = 'GZ = 6-12%',

font.title = c(20, "bold"),

xlab = NULL,

ylab = NULL,

legend = "none"

)

# 5%

splots[[3]] <- ggsurvplot(

methfit5,

data = df,

size = 1,

xscale = 365.25,

break.x.by = 365.25,

break.y.by = 0.5,

palette = c("#81C784","#0D47A1", "#B71C1C"),

conf.int = TRUE,

title = 'GZ = 5-12%',

font.title = c(20, "bold"),

font.y = c(18),

xlab = NULL,

ylab = 'Survival Probability',

legend = "none"

)

# 4%

splots[[7]] <- ggsurvplot(

methfit4,

data = df,

size = 1,

xscale = 365.25,

break.x.by = 365.25,

break.y.by = 0.5,

palette = c("#81C784","#0D47A1", "#B71C1C"),

conf.int = TRUE,

title = 'GZ = 4-12%',

font.title = c(20, "bold"),

xlab = NULL,

ylab = NULL,

legend = "none"

)

# 3%

splots[[11]] <- ggsurvplot(

methfit3,

data = df,

size = 1,

xscale = 365.25,

break.x.by = 365.25,

break.y.by = 0.5,

palette = c("#81C784","#0D47A1", "#B71C1C"),

conf.int = TRUE,

title = 'GZ = 3-12%',

font.title = c(20, "bold"),

xlab = NULL,

ylab = NULL,

legend = "none"

)

# 2%

splots[[4]] <- ggsurvplot(

methfit2,

data = df,

size = 1,

xscale = 365.25,

break.x.by = 365.25,

break.y.by = 0.5,

palette = c("#81C784","#0D47A1", "#B71C1C"),

conf.int = TRUE,

title = 'GZ = 2-12%',

xlab = "Time (Years)",

ylab = 'Survival Probability',

font.title = c(20, "bold"),

font.y = c(18),

font.x = c(18),

legend = "none"

)

# 1%

splots[[8]] <- ggsurvplot(

methfit1,

data = df,

size = 1,

xscale = 365.25,

break.x.by = 365.25,

break.y.by = 0.5,

palette = c("#81C784","#0D47A1", "#B71C1C"),

conf.int = TRUE,

title = 'GZ = 1-12%',

ylab = NULL,

legend = "none",

xlab = "Time (Years)",

font.title = c(20, "bold"),

font.x = c(18)

# legend.labs =

# c("Methylated","Grey Zone","Unmethylated"),

# legend = "bottom"

)

# No GZ

splots[[12]] <- ggsurvplot(

methfit_no_GZ,

data = df,

size = 1,

xscale = 365.25,

break.x.by = 365.25,

break.y.by = 0.5,

palette = c("#0D47A1", "#B71C1C"),

conf.int = TRUE,

title = 'No GZ',

xlab = "Time (Years)",

ylab = NULL,

legend = "none",

font.title = c(20, "bold"),

font.x = c(18)

)

# Arrange ggsurvplots and save figure

surv_plots <- arrange_ggsurvplots(splots, print = TRUE, ncol = 3, nrow = 4)

surv_plots %>% ggsave(device="png", filename="Gray_Zone_Survivals.png", width = 15, height = 15, units = "in")

## Cox Modelling ---------------------------------------------------------------------

# Alter factor levels so that the grey zone is the reference level

df <- df %>%

mutate(

Methylation_Status_No_GZ = factor(Methylation_Status_No_GZ, levels = c("Methylated", "Unmethylated"))

, Methylation_Status_11 = factor(Methylation_Status_11, levels = c("Grey Zone", "Methylated", "Unmethylated"))

, Methylation_Status_10 = factor(Methylation_Status_10, levels = c("Grey Zone", "Methylated", "Unmethylated"))

, Methylation_Status_9 = factor(Methylation_Status_9, levels = c("Grey Zone", "Methylated", "Unmethylated"))

, Methylation_Status_8 = factor(Methylation_Status_8, levels = c("Grey Zone", "Methylated", "Unmethylated"))

, Methylation_Status_7 = factor(Methylation_Status_7, levels = c("Grey Zone", "Methylated", "Unmethylated"))

, Methylation_Status_6 = factor(Methylation_Status_6, levels = c("Grey Zone", "Methylated", "Unmethylated"))

, Methylation_Status_5 = factor(Methylation_Status_5, levels = c("Grey Zone", "Methylated", "Unmethylated"))

, Methylation_Status_4 = factor(Methylation_Status_4, levels = c("Grey Zone", "Methylated", "Unmethylated"))

, Methylation_Status_3 = factor(Methylation_Status_3, levels = c("Grey Zone", "Methylated", "Unmethylated"))

, Methylation_Status_2 = factor(Methylation_Status_2, levels = c("Grey Zone", "Methylated", "Unmethylated"))

, Methylation_Status_1 = factor(Methylation_Status_1, levels = c("Grey Zone", "Methylated", "Unmethylated"))

)

# Hazard ratios and p values for each univariate Cox Proportional Hazards model

summary(coxph(Surv(Survival_Days, Died) ~ Methylation_Status_No_GZ, data = df))

# summary(coxph(Surv(Survival_Days, Died) ~ Methylation_Status_11, data = df))

lapply(c("Methylation_Status_11","Methylation_Status_10","Methylation_Status_9","Methylation_Status_8","Methylation_Status_7","Methylation_Status_6","Methylation_Status_5","Methylation_Status_4","Methylation_Status_3","Methylation_Status_2","Methylation_Status_1"),

function(x) {

formula <- as.formula(paste('Surv(Survival_Days, Died)~ ',as.factor(x)))

summary(coxph(formula, data = df))

coxFit <- summary(coxph(formula, data = df))

merge(coxFit$conf.int[,c("exp(coef)","lower .95","upper .95")],coxFit$coefficients[,c("exp(coef)","Pr(>|z|)")],

by=c("row.names","exp(coef)"),

all.x=TRUE)

# coxFit$logtest # log likelihood ratio test

}) # exp(coef) = hazard ratio, p value corresponds to hypothesis that the hazard ratio != 1

### Multivariate analysis ------------------------------------------------------

## Age, Sex and methylation status

summary(coxph(Surv(Survival_Days, Died) ~ Sex + Age_Diagnosis + Mean_methylation, data = df)) # Continuous

# summary(coxph(Surv(Survival_Days, Died) ~ Sex + Age_65_threshold + Methylation_Status_11, data = df))

lapply(c("Mean_methylation","Methylation_Status_No_GZ","Methylation_Status_11","Methylation_Status_10","Methylation_Status_9","Methylation_Status_8","Methylation_Status_7","Methylation_Status_6","Methylation_Status_5","Methylation_Status_4","Methylation_Status_3","Methylation_Status_2","Methylation_Status_1"),

function(x) {

formula <- as.formula(paste('Surv(Survival_Days, Died)~ Sex + Age_65_threshold +',as.factor(x)))

coxFit <- summary(coxph(formula, data = df))

merge(coxFit$conf.int[,c("exp(coef)","lower .95","upper .95")],coxFit$coefficients[,c("exp(coef)","Pr(>|z|)")],

by=c("row.names","exp(coef)"),

all.x=TRUE)

# coxFit$logtest # log likelihood ratio test

})

## Age and methylation status - hazard ratio and CIs

summary(coxph(Surv(Survival_Days, Died) ~ Age_Diagnosis + Mean_methylation, data = df)) # Continuous

lapply(c("Mean_methylation","Methylation_Status_No_GZ","Methylation_Status_11","Methylation_Status_10","Methylation_Status_9","Methylation_Status_8","Methylation_Status_7","Methylation_Status_6","Methylation_Status_5","Methylation_Status_4","Methylation_Status_3","Methylation_Status_2","Methylation_Status_1"),

function(x) {

formula <- as.formula(paste('Surv(Survival_Days, Died)~ Age_65_threshold +',as.factor(x)))

coxFit <- summary(coxph(formula, data = df))

merge(coxFit$conf.int[,c("exp(coef)","lower .95","upper .95")],coxFit$coefficients[,c("exp(coef)","Pr(>|z|)")],

by=c("row.names","exp(coef)"),

all.x=TRUE)

})

## Age and methylation status - Log likelihood ratio test

lapply(c("Mean_methylation","Methylation_Status_No_GZ","Methylation_Status_11","Methylation_Status_10","Methylation_Status_9","Methylation_Status_8","Methylation_Status_7","Methylation_Status_6","Methylation_Status_5","Methylation_Status_4","Methylation_Status_3","Methylation_Status_2","Methylation_Status_1"),

function(x) {

formula <- as.formula(paste('Surv(Survival_Days, Died)~ Age_65_threshold +',as.factor(x)))

coxFit <- summary(coxph(formula, data = df))

coxFit$logtest # log likelihood ratio test

})

# Likelihood ratio test - Compare the absence and presence of a gray zone

no_gz_fit <- coxph(Surv(Survival_Days, Died) ~ Age_65_threshold + Methylation_Status_No_GZ, data = df)

with_gz_fit <- coxph(Surv(Survival_Days, Died) ~ Age_65_threshold + Methylation_Status_5, data = df) # Change Methylation_Status_5 to your chosen model. Instructions on how to choose is in the paper.

anova(no_gz_fit,with_gz_fit,test="Chisq")

# Calculate log likelihood test statistic = -2*log(likelihood score from simple model/likelihood score from more complicated model)

-2 * log(80.943 / 88.894)
